# Cancer-targeted photoimmunotherapy induces antitumor immunity and can be augmented by anti-PD-1 therapy for durable anticancer responses in an immunologically active murine tumor model

**DOI:** 10.1007/s00262-022-03239-9

**Published:** 2022-07-01

**Authors:** Michelle A. Hsu, Stephanie M. Okamura, C. Daniel De Magalhaes Filho, Daniele M. Bergeron, Ahiram Rodriguez, Melissa West, Deepak Yadav, Roger Heim, Jerry J. Fong, Miguel Garcia-Guzman

**Affiliations:** Rakuten Medical, Inc., Translational Sciences, 11080 Roselle Street, San Diego, CA 92121 USA

**Keywords:** Photoimmunotherapy, Immunology, Immuno-oncology, Cancer

## Abstract

The complex immunosuppressive nature of solid tumor microenvironments poses a significant challenge to generating efficacious and durable anticancer responses. Photoimmunotherapy is a cancer treatment strategy by which an antibody is conjugated with a non-toxic light-activatable dye. Following administration of the conjugate and binding to the target tumor, subsequent local laser illumination activates the dye, resulting in highly specific target cell membrane disruption. Here we demonstrate that photoimmunotherapy treatment elicited tumor necrosis, thus inducing immunogenic cell death characterized by the release of damage-associated molecular patterns (DAMPs). Photoimmunotherapy-killed tumor cells activated dendritic cells (DC), leading to the production of proinflammatory cytokines, T cell stimulation, priming antigen-specific T cells, and durable memory T cell responses, which led complete responder mice to effectively reject new tumors upon rechallenge. PD-1 blockade in combination with photoimmunotherapy enhanced overall anticancer efficacy, including against anti-PD-1-resistant tumors. The combination treatment also elicited abscopal anticancer activity, as observed by reduction of distal, non-illuminated tumors, further demonstrating the ability of photoimmunotherapy to harness local and peripheral T cell responses. With this work we therefore delineate the immune mechanisms of action for photoimmunotherapy and demonstrate the potential for cancer-targeted photoimmunotherapy to be combined with other immunotherapy approaches for augmented, durable anticancer efficacy. Moreover, we demonstrate responses utilizing various immunocompetent mouse models, as well as in vitro data from human cells, suggesting broad translational potential.

**Supplementary Information:**

The online version contains supplementary material available at 10.1007/s00262-022-03239-9.

## Introduction

Despite the revolutionary addition of immune checkpoint inhibitor (ICI) therapies to cancer treatments [1, 2], the development of strategies to successfully ablate solid tumors remains challenging due to several factors, including the complex immunosuppressive nature of the tumor microenvironment (TME). The TME represents a dysfunctional immunologic niche characterized by a network of immunosuppressive innate and adaptive immune cells, amongst tumor-supportive fibroblasts, endothelial cells, and extracellular matrix proteins, posing a hurdle for T cells to encounter tumor antigens and mount effector responses [3, 4]. Therapeutic strategies that may augment cancer neoantigen exposure to elicit durable anticancer immune responses are therefore highly desirable [3–7].

Photoimmunotherapy is a unique treatment approach by which an antibody, specific to an antigen expressed on target cells, is conjugated with a light-activatable dye to localize the dye to the targeted cell. Systemically administered tumor antigen-targeting antibody-dye conjugates bind directly to tumor cells and accumulate within the tumor lesion. After tumor binding, localized laser illumination with nonthermal red light (690 nm) activates the dye and results in tumor cell membrane disruption and local tumor-specific cell killing with minimal damage to surrounding healthy tissue [8–16]. The original studies applying photoimmunotherapy treatment for cancer used antibodies conjugated to hematoporphyrin as the light-activatable dye, and subsequent studies demonstrated that various photosensitizing dyes may be conjugated as well [17–22]. More recently, photoimmunotherapy treatment utilizing antibodies conjugated to the non-toxic dye IRDye® 700DX (IR700), which is activated with 690 nm nonthermal red light, has been demonstrated to be efficacious in preclinical models, and overcomes some of the shortcomings of previously used dyes [9, 10].

Photoimmunotherapy encompasses key advantages over other treatment options due to the localized tumor treatment that minimizes damage to normal, surrounding tissues. Because the IR700 dye is non-toxic without light activation, the antibody-IR700 conjugate will not induce toxicities in distal, non-illuminated healthy tissues as encountered for most systemic therapies. Cancer treatment modalities such as radiotherapy or chemotherapy may compromise host immunity due to collateral toxicity towards antitumor immune cells, but photoimmunotherapy stimulates antitumor immunity by inducing immunogenic cell death (ICD). ICD results in the release of damage-associated molecular pattern (DAMP) molecules which activate dendritic cells (DCs) and facilitate the generation of potent tumor-neoantigen specific T cell responses [16, 23]. Rapid photoimmunotherapy-induced cell necrosis compromises the structural integrity of the TME, likely facilitating access for newly primed and/or reinvigorated tumor-specific T cells to exert effector functions. Finally, photoimmunotherapy may circumvent common mechanisms of resistance due to its cell signaling-independent mechanism of action and ability to target a diverse repertoire of tumor antigens [8–12, 16, 24]. Indeed, tumor killing by photodynamic therapy (PDT), a similar method of treatment utilizing an unconjugated photosensitizer combined with tumor-specific illumination, has been observed to enhance the antitumor immune response in preclinical models [25–28]. For example, many studies have demonstrated that PDT treatment resulted in the release of the various DAMPs to activate innate immune cells [29, 30]. Furthermore, PDT treatment activated dendritic cells to engulf PDT-killed tumor cells, produce pro-inflammatory cytokines, and migrate to lymph nodes to activate cytolytic immune cells.

The studies described here support and expand on the previous findings that photoimmunotherapy treatment induced rapid necrosis of tumor cells which exhibited hallmarks of ICD, leading to DC maturation and T cell activation within the TME. Moreover, photoimmunotherapy elicited durable peripheral tumor-specific memory T cell responses, and successfully ablated newly inoculated tumor cells upon syngeneic rechallenge in mice. PD-1 blockade acts to reinvigorate chronically activated T cells within the TME and draining lymph nodes [31]. Here, the addition of anti-PD-1 antibody treatment with photoimmunotherapy augmented the anti-cancer responses, further supporting an immune-activating mechanism for cancer-targeted photoimmunotherapy in vivo, as well as demonstrating the utility of photoimmunotherapy in combination with immunomodulators. Finally, the combination of photoimmunotherapy with anti-PD-1 treatment also elicited abscopal effects at distal, non-illuminated tumor sites—a mechanism which is highly desirable for improving treatment efficacy and durability for distant metastases and in advanced disease cases.

## Methods

### IR700 conjugation to anti-EphA2 antibody

Anti-EphA2 antibody (Absolute Antibody, Cat. No. Ab00430-1.1), or IgG1 isotype control (BioXCell, Cat. No. BE0083), was buffer-exchanged into sterile PBS by centrifugation at 3220 × *g* for 16 min, 4ºC, through an Amicon Ultra-15 30 kDa filter unit five times. After the last centrifugation, 3X volume of sterile, ice cold 100 mM phosphate buffer pH 9.0 was added to the antibody in PBS. Frozen, lyophilized IRDye 700DX-NHS Ester (procured from either LI-COR, Cat. No. 929–70,011; or Irix Pharmaceuticals, special order product) was reconstituted in DMSO to achieve a 10 mg/ml solution and kept in the dark until ready for use. IRDye 700DX-NHS-DMSO solution was added to the antibody solution prepared above at molar ratios (IR700:antibody) of: 8:1 (anti-EphA2-IR700), or 6.3:1 (anti-mouse IgG1 isotype control). After incubation at 25 °C for 2 h in the dark, the conjugation reaction was quenched by the addition of sterile 1 M glycine pH 8.5 and exchanged into sterile PBS by centrifugation through an Amicon Ultra-15 30 kDa filter unit 5 to 9 times, depending on amount of antibody conjugated. Antibody concentration and final dye-to-antibody ratio was determined by high-performance liquid chromatography (HPLC) with separation by size exclusion columns and quantified by UV absorbance at 280 nm (for antibody) and 690 nm (for IR700).

### Cell lines and culture conditions

A431 (ATCC CRL-1555) and BxPC-3 (ATCC CRL-1687) tumor cells (and lentiviral transduced CT26-EphA2, 4T1-EpCam and LL/2-EphA2 cell lines, as described below) were grown in complete RPMI [containing 10% fetal bovine serum (FBS) and 1% penicillin/streptomycin (P/S)]. FaDu cells (ATCC HTB-43) were cultured in complete EMEM (containing 10% FBS and 1% P/S). Cells were maintained at 37 °C and 5% CO_2_.

### Lentiviral transduction

To create CT26-EphA2 cells, murine Ephrin A2 lentiviral particles containing the puromycin resistance gene (Genecopoeia, Cat. No. LPP-CS-Mm02340-Lv225-03–300) were added to wild-type CT26 cell (ATCC Cat. No. CRL-2638) cultures at a multiplicity of infection of 20:1 and incubated overnight. Culture media containing the lentiviral particles was then removed and cells were cultured for an additional 4 days at which time puromycin (InvivoGen, Cat. No. ant-pr-5) was added to create antibiotic selection. Puromycin selection was discontinued once a healthy population of cells stabilized and returned to typical growth patterns in the presence of the antibiotic. Transduced CT26-EphA2 cells were then sorted into individual wells and selected based on growth rate and EphA2 expression. These cells were clonally expanded and cryopreserved for future use. Prior to use, expression of EphA2 was confirmed by flow cytometry using anti-EphA2-IR700 versus IgG1-IR700 isotype control. Similarly, 4T1-EpCam cells were generated from 4T1 cells (ATCC Cat. No. CRL-2539) and LL/2-EphA2 cells were generated from LL/2 cells (ATCC, Cat No. CRL-1642) via transduction with lentiviral particles (Genecopoeia; Cat. No. LPP-CS-Mm02340-Lv225-03–300) and cultured in complete RPMI containing 2 μg/ml puromycin.

### Cetuximab-IR700 binding assay to BxPC-3 cells

Cultured BxPC-3 cells were resuspended in 1 mL FACS sorting buffer (PBS, 20 mM HEPES, 1 mM EDTA, 1% BSA) containing 1 µg/mL cetuximab-IR700 and incubated 1 h at room temperature. Unbound reagent was removed by washing, and cells were fixed prior to acquisition. To demonstrate specificity, 100 µg/mL cetuximab (Erbitux, Eli Lilly, lot #C1500202) was included as an unlabeled competitor during staining with cetuximab-IR700. Unstained cells were treated with buffer only. Samples were acquired on an Attune® Acoustic Focusing Flow Cytometer (Life Technologies).

### In vitro photoimmunotherapy of CT26-EphA2 or BxPC-3 cells

To evaluate the sensitivity of the CT26-EphA2 clonal line to in vitro photoimmunotherapy, cells were exposed to a threefold, 7-point, serial dilution of anti-EphA2-IR700 conjugate starting at 1 µg/ml in triplicate. In other experiments BxPC-3 cells were similarly treated with cetuximab-IR700 at indicated antibody-conjugate and light doses. Cells were incubated with conjugate at 37ºC, 5% CO_2_ for one hour, then treated with 690 nm red light at a density of 150 mW/cm^2^, at twofold increments of doses from 0 to 16 J/cm^2^ for CT26-EphA2 cells or 0 to 64 J/cm^2^ for BxPC-3 cells using a custom laser device (co-developed by Rakuten Medical Inc and Omicron (Germany) to facilitate uniform illumination with 690 nm light across a 96-well assay plate). After light treatment, culture media were replaced with complete growth medium containing Cell Tox Green (Promega, Cat. No. 8731), including two cell free wells for background fluorescence calculations, and incubated at 37ºC, 5% CO_2_ for 24 h. Fluorescence in each well, an indicator of membrane integrity and therefore cytotoxicity, was measured on a SpectraMax Gemini or M5 plate reader (Molecular Devices). After this first “pre-lysis” read, the cells were fully lysed with the addition of 5 µL/well of a 60:40 mix of complete growth medium and lysis buffer (Promega Cat. No. G182A) (including cell free wells). Plates were incubated with lysis buffer for 30 min at 37ºC, 5% CO_2_, and fluorescence was read again as a “post-lysis” read. Percent cell death for each well was then calculated by subtracting the average cell-free well fluorescence value from each well of each read, then calculating the ratio of background corrected pre-lysis read to background corrected post-lysis read. Percent cell death values for replicate wells were analyzed using GraphPad Prism v7.0c (GraphPad Software) using a 4-parameter non-linear curve fit, log(agonist) vs. response, and EC_50_ values were calculated.

### Flow cytometric detection of ICD markers from in vitro cultured A431 and FaDu cells

A431 or FaDu cells were harvested using HyQtase detachment solution and plated at 10,000 cells/100 μL/well in a 96 well plate. The cells were incubated for one hour at 37 °C with 100 μL of serum free medium only or 500 ng/mL cetuximab-IR700 in serum free medium. Cells were then either illuminated with 6 J/cm^2^ 690 nm light (A431), 12 J/cm^2^ 690 nm light (FaDu) or no light, as described above. Treated cells were then stained for flow cytometry. Approximately 40,000 cells/test (pooled from 4 treated wells) were centrifuged at 900 rpm for six minutes and resuspended in 100 μL flow cytometry buffer (PBS containing 1% BSA and 0.01% sodium azide). Cells were then incubated at room temperature for 25 min with fluorescently labeled anti-HSP90 (Enzo Cat. No. ADI-SPA-830PE-F), anti-HSP70 (Enzo Cat. No. ADI-SPA-810–488-F), or anti-CRT antibodies (Enzo Cat. No. ADI-SPA-601–488-D). Cells were then washed and incubated for 5 to 10 min at room temperature in flow cytometry buffer containing 7-AAD viability staining solution (Biolegend Cat. No. 79993) prior to acquisition on an Attune® Acoustic Focusing Flow Cytometer (Life Technologies) under high sensitivity mode within an hour of staining. At least 10,000 events were collected. Flow cytometry data were analyzed using Attune® Cytometric Software. Cell were gated first by forward and side scatter, then 7-AAD negative live cells followed by evaluation of ICD markers.

### In vitro determination of HMGB1

A431 or FaDu cells were plated at 10,000 cells/100 μL /well in a 96 well plate. The following day, the cells were incubated for one hour at 37 °C with 100 μL of 500 ng/mL cetuximab-IR700 in serum free medium. The cetuximab-IR700 treated cells were then illuminated with 32 J/cm^2 ^of 690 nm light at 150 mW/cm^2^ (or no applied light as control), as described above. The culture supernatant was collected 1 h after photoimmunotherapy, centrifuged at 5000x*g* for three minutes to remove debris, and stored at -20 °C for subsequent analysis. The HMGB1 ELISA (IBL, Cat. No. ST51011) was performed per manufacturer’s protocol. Briefly, a calibration standard curve was prepared by diluting HMGB1 standard stock in diluent buffer, then serially diluting for a total of 6 points (80 ng/ml to 2.5 ng/ml). Samples or controls were added to each well, then the plate was sealed and incubated overnight at 37 °C. Plates were then washed and incubated with enzyme conjugate for 2 h at room temperature. Excess enzyme conjugate was washed off and colorimetric development buffer was added for 30 min at room temperature. The reaction was stopped by adding 100 μL/well of stop solution. Absorbance at 450 nm was measured on a SpectraMax Plus plate reader (Molecular Devices). The HMGB1 standard curve was graphed with a 4-parameter logistics fit and the test sample data interpolated against the standard curve to determine HMGB1 concentration in each sample.

### ATP release assay

Indicated cell lines were plated at 40,000/well in a 96-well plate. After 24 h, the conjugate (1 μg/ml) was added to the conjugate only and photoimmunotherapy wells. Approximately 2 h after conjugate administration, photoimmunotherapy wells were dosed with 16 J/cm^2^ of light, as described above. Supernatant was harvested at 1, 3, 6, 26, and 51 h post-light administration and frozen at -20 °C. ATP concentrations were detected using the Sigma Aldrich Adenosine 5’-triphosphate (ATP) Bioluminescent Assay Kit (Sigma Aldrich Cat. No. FLAA-1KT). A 1:300 dilution of ATP Assay Mix was used. Luminescence was detected using a Spectramax M5 plate reader. Percent cell death was measured using the CellTox Green assay (Promega, Cat. No. G8741) following manufacturer recommendations.

### Type I interferons and ANXA1 quantification assays

Cultured A431, BxPC-3 or FaDu cells were treated with cetuximab-IR700 photoimmunotherapy or left untreated. At indicated times after photoimmunotherapy, supernatants were collected and assayed for detection of human ANXA1 (24 h), IFN-α, and IFN-β (3 h) using the Meso Scale Discovery (MSD) platform (MESO QuickPlex SQ 120 instrument). Samples were assayed according to the manufacturer’s recommendations using the R-plex kit for ANXA1 (MSD; Cat. No. F21YK) and U-plex kits for IFN-α2α and IFN-β (MSD; Cat. Nos. K151VHK and B21VI, respectively).

### Tumor:iDC co-cultures

Photoimmunotherapy-killed or untreated (negative control) FaDu cells were co-cultured with primary iDCs derived from 4 individual healthy human donors (Astarte Biologics Cat. No. 1010) in complete RPMI for 2 days at 37 °C for flow cytometry analysis and cytokine analysis. iDCs were prepared by culturing enriched primary monocytes in the presence of IL-4 and GM-CSF, as described by the manufacturer. Positive control cells were cultured with lipopolysaccharide (5 μg/mL) (eBioscience, Cat. No. 00–4976-93) for 24 h. After indicated times, supernatants were collected for cytokine analysis by Luminex. For flow cytometry, cells were gently detached and stained as described below.

### Cytokine release from dendritic cells

Culture supernatants from iDC co-culture experiments were sent to eBiosciences (Thermo Fisher) for analysis of TNF-α, IP-10, IL-1β, MIP-1α, MIP-1β, and IL-8 using the Luminex platform. Samples were run in triplicate both as neat and at 1:5 dilution. Culture media was run as negative control and exhibited values lower than detection limit of the assay for all the analytes.

### In vivo tumor implant and anti-PD-1 treatment 

Animal studies were carried out in compliance with NIH guidelines for the care and use of laboratory animals. Female BALB/c or C57BL/6 mice (5 to 8 weeks old) were purchased from Charles River Laboratories and allowed to acclimate prior to experimental enrollment. Following the acclimation period, animals were implanted in the right hind flank with 1 to 3 × 10^6^ CT26-EphA2 cells or 5 × 10^5^ MCA205-EphaA2 cells after dissociation with Accutase (Corning, Cat. No. 25–058-Cl), centrifugation, and resuspension in a 1:1 mix of RPMI-1640 medium and Matrigel (Corning, Cat. No. 354234). In some experiments mice that were identified as complete responders were rechallenged with either 3 × 10^6^ CT26-EphA2 or 1 × 10^6^ 4T1 tumor cells in the opposite hind flank or axilla, respectively. Tumor growth was monitored with caliper measurements wherein tumor volume was calculated using the industry standard equation: (L x W^2^)/2, where L equals the tumor measurement in the longest dimension, and W equals the measurement in the shortest dimension. Animals were randomized and sorted into treatment groups upon reaching tumor enrollment size (average 150 mm^3^). In some experiments mice received 100 μg anti-CD40L (BioXCell, clone MR-1, Cat. No. BP0017-1), 100 μg (CT26) or 10 mg/kg (MCA205) anti-PD-1 (BioXCell, clone RMP1-14, Cat. No. BP0146), or 100 μg anti-CD8α (BioXCell, clone 2.43, Cat. No. BP0061) intraperitoneally (i.p.) at indicated time points. All tumor growth studies and tumor rechallenge studies were performed at least n = 2 replicates by different personnel with similar results.

### In vivo photoimmunotherapy

Tumor inoculated mice were anesthetized with 2% isoflurane in oxygen and dosed with 100 µL antibody-conjugate (anti-EphA2-IR700 or cetuximab-IR700), diluted to 1 mg/ml in PBS via intravenous administration through the retro-orbital (RO) plexus. For animals that received photoimmunotherapy treatment, tumors were exposed to 100 or 150 J/cm^2^ of 690 nm light at a power density of 150 mW/cm^2^ at 24 ± 2 h following antibody-conjugate administration, or no light for control conditions. Briefly, animals were anesthetized with 2% isoflurane in oxygen, delivered through a non-rebreathing nose cone. Body temperature was maintained with warming lamps, and ophthalmic eye ointment was placed on both eyes to prevent drying. Animal’s bodies were shielded with matte black aluminum foil and foil tape, with a small opening placed directly over the subcutaneous tumor. Laser light of 690 nm wavelength was supplied by an MDL-III-690–800 mW laser equipped with fiber coupling, collimator and beam expander to produce a parallel light beam. Lasers were turned on a minimum of 30 min before use to stabilize the light output. Light output was adjusted using an optical power meter to 105 mW ± 5 mW, equivalent to 150 mW/cm^2^ ± 7 mW/cm^2^. Laser light was placed directly over the tumor for 11 min, 6 s, delivering 100 J/cm^2^ light. Animals were monitored for respiratory rate and isoflurane was adjusted accordingly. Following photoimmunotherapy, animals were allowed to recover in their cages until fully alert and recumbent. Complete responses were defined as lack of palpable tumor.

IR700 fluorescence imaging was performed using an AMI imager (Spectral Instruments Imaging) at the indicated time points to confirm and evaluate accumulation of the conjugate in the tumor. Mice were anesthetized with 2% isoflurane in oxygen and placed in the imaging chamber, supplied with a 5-cone manifold, on a heated imaging stand for body temperature maintenance. For fluorescent imaging, images were acquired at 675 nm excitation and 730 nm emission wavelengths, with no binning and 120 to 180 s exposure time. Images were analyzed with AMI View software version 1.7.06, by placing circular Regions of Interest (ROIs) over the tumor and measuring radiance in photons/second.

### Tumor viability measured by bioluminescence

BxPC-3-luciferase-expressing human pancreatic cell line, which stably express luciferase (JCRB cell bank Cat. No. JCRB1448) were inoculated into the hind flanks of Hsd:Athymic Nude-*Foxn1*^nu^ mice (Envigo RMS Inc). Tumors were allowed to establish for 6 days, then treated with cetuximab-IR700 by retro-orbital injection (RO). Localized laser illumination at 50, 150, or 300 J/cm^2^ was applied 24 ± 1 h later. Bioluminescence imaging was conducted on an AMI imager (Spectral Instruments Imaging) at time 0 (pre-light), and 1 or 3 days after the application of light.

### Vaccinal effects

CT26-EphA2 cells were treated in vitro with 25 μM doxorubicin, 50 μM cisplatin, anti-EphA2-IR700, or EphA2 photoimmunotherapy. When approximately 50% of cells had died, as determined in a cell death kinetics assay, CT26-EphA2 cells were inoculated at a density of 7.5 × 10^6^ cells/ml (1.5 × 10^6^ cells/animal) in the right hind flank. Seven days after implant with dying cells, mice were implanted with 5 × 10^5^ healthy CT26-EphA2 cells in the opposite hind flank. Resultant tumors were measured twice weekly.

### Flow cytometry

Single cell suspensions from dissociated tumors were resuspended in Zombie NIR live/dead stain (Biolegend, Cat. No. 423106) and incubated for 15 to 30 min at room temperature. Cells were washed twice, then incubated in FcR block [BD Biosciences, Cat. No. 553142 (mouse) or 564,220 (human)], followed by antibody cocktails designed to stain activation patterns among T cells, NK cells, or DCs (see Supplementary Table 1) for 30 min on ice. For in vitro DC co-culture experiments, adherent cells were gently detached using enzyme-free cell dissociation buffer. Single-cell suspensions were stained with Zombie Green (Biolegend, Cat. No. 423111) for 10 to 15 min at room temperature in the dark. Cells were washed prior to the addition of surface antibodies and stained for 25 to 30 min at room temperature in the dark. Finally, cells were washed again prior to acquisition on an Attune® Acoustic Focusing Flow Cytometer (Life Technologies). Analysis was performed using Attune® Cytometric Software and gated based on forward and side scatter, singlets, and live dead, CD45 + cells. Further gates were then determined based on the population of interest and validated by isotype controls. In some analyses fold increase of median fluorescence intensity (MFI) was calculated by dividing the MFI after photoimmunotherapy by the MFI of anti-EphA2-IR700 without light.

### Cytotoxic T Lymphocyte assay

Briefly, mice were euthanized 2 weeks after photoimmunotherapy (18 days after tumor implant), and spleens were harvested. Splenocytes were cultured and expanded in vitro for four days in the presence of 40 µg/mL AH1 peptide (Anaspec, Cat. No. AS-64798), an antigen expressed on the surface of CT26 cells. After in vitro expansion, a titration of splenocytes was co-cultured with either target CT26-EphA2 cells or negative control target BxPC-3 cells (which do not express AH1) and assessed for their ability to kill the target tumor cells for 4 h. Cytotoxicity was determined by Lactic Acid Dehydrogenase (LDH) release (Promega, Cat. No. G1780), measured at 490 nm on a SpectraMax M5 plate reader (Molecular Devices).

### Statistical analysis

Most comparisons were calculated by GraphPad Prism software (v9.1.0) using unpaired t-tests, assuming parametric distribution, or two-way ANOVA. For cytokine analysis, statistics were calculated and provided by eBiosciences (Thermo Fisher).

## RESULTS

### Cetuximab-IR700 antibody-dye conjugate specifically binds EGFR on tumor cells and induces rapid cell death upon administration of applied light.

The binding specificity of cetuximab-IR700 dye conjugate (anti-EGFR-IR700 antibody conjugate; CTX-IR700) to epidermal growth factor receptor (EGFR) was evaluated in vitro on BxPC-3 pancreatic adenocarcinoma tumor cells by flow cytometry. CTX-IR700 bound to BxPC-3 cells in an EGFR-dependent manner, demonstrated by the abrogated signal upon the addition of an excess of unconjugated, competitive cetuximab (CTX) (Supplementary Fig. S1A). BxPC-3 cells exposed to CTX-IR700 with 690 nm red light exhibited conjugate dose-dependent and light dose-dependent cell death, whereas exposure to only CTX-IR700 without 690 nm red light, or vice versa, did not exhibit cell death above background levels, underscoring that both components of the two-part treatment are required for cytotoxicity (Supplementary Fig. S1B).

To evaluate CTX-IR700 photoimmunotherapy in vivo, nude mice subcutaneously implanted with BxPC-3-luciferase (BxPC-3-luc) tumor cells were evaluated for conjugate uptake into the tumor after a single systemic administration of CTX-IR700. IR700 fluorescence was detected within 1 h after administration and peaked at 24 h (**Supplementary Fig. S1C**). To determine whether CTX-IR700 photoimmunotherapy kills EGFR-expressing cells in vivo, the tumors of BxPC-3-luc-bearing nude mice received 690 nm red light 24 h after CTX-IR700 administration. CTX-IR700 photoimmunotherapy elicited a notable reduction in tumor viability after illumination in a light dose-dependent manner, as measured by bioluminescence (BLI) imaging (Supplementary Fig. S1D). As expected, the most notable reduction of tumor viability occurred one day post illumination, further supporting that the mechanism of cell killing by photoimmunotherapy treatment requires the combination of a cell bound antibody-IR700 conjugate with light illumination. Therefore, it was also not surprising to observe a trend towards tumor regrowth at day 3 post light treatment by BLI, especially in immunocompromised mice. Control mice receiving CTX-IR700 in the absence of applied light did not exhibit tumor reduction [9, 16, 24, 32]. These results demonstrate the binding specificity and direct tumor cell killing activities of CTX-IR700 photoimmunotherapy in vitro and in vivo.

### In vitro CTX-IR700 photoimmunotherapy induces immunogenic cell death

Photoimmunotherapy treatment disrupts the targeted cell membrane, resulting in rapid tumor necrosis. Due to this mechanism, photoimmunotherapy has been demonstrated to induce some markers of ICD, thereby potentially activating durable T cell responses to newly exposed cancer neoantigens [15, 23, 33, 34]. To further characterize inflammatory ICD markers induced by photoimmunotherapy, EGFR-expressing human carcinoma cell lines A431 and FaDu were treated with CTX-IR700 photoimmunotherapy and assessed for the expression or release of DAMP molecules including Hsp70, Hsp90, calreticulin (CRT), ATP, and High Mobility Group Protein B1 (HMGB1) [35–38]. In agreement with the data of others [10, 16], CTX-IR700 photoimmunotherapy-treated carcinoma cells demonstrated an apparent increase in surface expression of Hsp70, Hsp90, and CRT, as observed by flow cytometry (Supplementary Fig. S2A). Similarly, increased concentrations of soluble HMGB1, which promotes DC activation, were detected in culture supernatants (Supplementary Fig. S2B). Photoimmunotherapy treatment has been demonstrated to induce the rapid release of ATP, consistent with ICD [16, 39]. Here, ATP release was observed following photoimmunotherapy treatment in multiple carcinoma cell lines from human or mouse origin with different target antigens (Supplementary Fig. S2C). Moreover, in all tumor lines tested, the peak of ATP release occurred within 2 to 6 h following photoimmunotherapy treatment but preceding maximal cell death, suggesting that ATP was actively transported into the extracellular space, rather than passively diffused across perturbed membranes of dead cells (Supplementary Fig. S2D). Finally, DC maturation molecules Annexin A1, IFN-α2a, and IFN-β were also secreted from photoimmunotherapy-treated cells (Supplementary Fig. S2E and S2F) [4, 40]. Specifically, photoimmunotherapy-treated A431 cells abundantly secreted Annexin A1 and both type I interferons, whereas BxPC-3 and FaDu cells secreted only Annexin A1. Together, these data demonstrate that photoimmunotherapy treatment resulted in the rapid release and upregulation of surface expression of multiple ICD molecules across multiple tumor cell lines and species.

### DCs exposed to photoimmunotherapy-killed cells become activated and produce pro-inflammatory cytokines

Dendritic cells are tumor-resident, innate immune cells that are directly activated in response to ICD molecules. Primary monocyte derived dendritic cells (MDDC) from four individual donors were co-cultured with either photoimmunotherapy-exposed FaDu cells or naïve FaDu cells for 48 h to evaluate innate immune activation. DCs exposed to photoimmunotherapy-treated FaDu cells significantly upregulated expression of the co-stimulatory molecule CD86 and trended toward an increase in MHC II, in comparison to DCs co-cultured with naïve FaDu cells (Supplementary Fig. S3A). Consistent with surface molecule expression, DCs exposed to photoimmunotherapy-killed FaDu cells produced significantly higher concentrations of pro-inflammatory cytokines including TNF-α, IL-1β, MIP-1α, MIP-1β and IL-8, and a trend toward increased IP-10, as compared with controls (Supplementary Fig. S3B). Together, our data demonstrated that CTX-IR700 photoimmunotherapy successfully kills tumor cells in an antigen-specific manner, resulting in immunogenic cell death and activation of DCs in vitro.

### Characterization of an immunocompetent mouse tumor model for targeted photoimmunotherapy

**Fig. 1:**
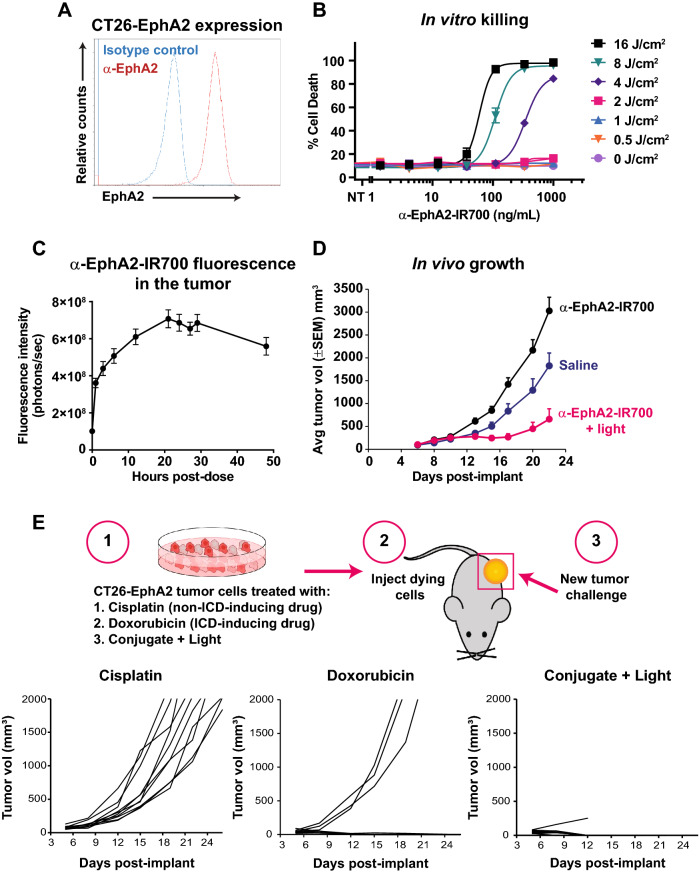
Characterization of an immunocompetent mouse tumor model for cancer-targeted photoimmunotherapy. Flow cytometry analysis demonstrated stable expression of mouse EphA2 on the surface of CT26 mouse colon carcinoma cells after viral transduction (**A**). CT26-EphA2 cells were treated with increasing concentrations of anti-EphA2-IR700 followed by illumination at indicated doses, demonstrating that EphA2 photoimmunotherapy results in cell killing in a light- and conjugate- dose-dependent manner in vitro (**B**). Anti-EphA2-IR700 accumulates in CT26-EphA2 tumors in mice after systemic administration, as detected by fluorescence imaging (**C**). Mice were implanted with CT26-EphA2 tumors, then treated with anti-EphA2-IR700 and light or only anti-EphA2-IR700 without light, as reference. Tumor growth was inhibited in the anti-EphA2-IR700 plus light group (n = 15 per group). Final tumor measurements: saline vs EphA2 photoimmunotherapy, p < 0.01; EphA2 vs EphA2 photoimmunotherapy, p < 0.0001 based on two-way ANOVA and Tukey test. The tumor growth results shown are representative of several replicate experiments performed by different personnel. (**D**). The ability of EphA2 photoimmunotherapy to elicit vaccinal effects as compared to known ICD-inducing or non-ICD-inducing conditions was determined. Cells killed with cisplatin, doxorubicin, or anti-EphA2-IR700 plus light were allowed to achieve 50% cell death, then were implanted into the right hind flank of naïve mice. Seven days after implant with dying cells, mice were challenged with viable CT26-EphA2 cells in the opposite hind flank and tumor growth was measured for 21 days (**E**)

In order to determine the immunomodulatory activity of cancer-targeted photoimmunotherapy in vivo, a syngeneic, subcutaneous mouse model with CT26 mouse colon carcinoma cells was developed. CT26 tumors were chosen as a model system due to its immunologically active tumor microenvironment, as demonstrated by its responsiveness to checkpoint inhibition and harboring both functional cytotoxic lymphocytes [41–43]. CT26 cells were transduced with a lentiviral vector to stably express EphA2 (hereafter referred to as CT26-EphA2 cells) that could be targeted with an anti-EphA2-IR700 antibody conjugate. Binding of the conjugate to the recombinant cells was confirmed by flow cytometry (Fig. 1A). To evaluate this system for photoimmunotherapy, CT26-EphA2 cells were incubated in vitro with anti-EphA2-IR700 and treated with 690 nm light. Cell death was observed in a conjugate dose and light dose-dependent manner (Fig. 1B).

CT26-EphA2 cells were therefore implanted into the hind flank of allogenic BALB/c mice to establish tumors. Once the tumors had reached palpable size (averaging 150 mm^2^), anti-EphA2-IR700 dye conjugate was administered and tracked for accumulation within the tumor by fluorescence imaging over 48 h post administration (Fig. 1C). Anti-EphA2-IR700 conjugate reached maximum uptake in CT26-EphA2 tumors approximately 20 h after administration and began to decline after 28 h post dose. Subsequently, mice bearing subcutaneous CT26-EphA2 tumors were administered anti-EphA2-IR700 alone or followed by 690 nm illumination of the tumor 24 h later (photoimmunotherapy treatment). Control mice received either saline or anti-EphA2-IR700 without light. Mice that received EphA2 photoimmunotherapy treatment exhibited notable tumor growth inhibition in comparison with the control animals. Unexpectedly, mice that received anti-EphA2-IR700 without light exhibited slightly enhanced tumor growth in comparison to mice that received only saline, due to unknown mechanisms. Therefore, the most appropriate control group was mice that received anti-EphA2-IR700 without applied light to differentiate between responses induced by the antibody-conjugate alone compared to photoimmunotherapy treatment (Fig. 1D).

### Extracorporeal EphA2 photoimmunotherapy treatment generates vaccinal effects against new tumor challenges

Therapeutic strategies that induce ICD to enhance adaptive immune cell priming may induce a durable vaccinal effect in vivo [44]. CT26-EphA2 tumor cells were treated in vitro with 1) the non-ICD-inducing agent cisplatin; 2) the ICD-inducing agent doxorubicin and 3) ICD-inducing photoimmunotherapy. When approximately 50% of cells had died, as determined by a cell death kinetics assay, the treated cells were subsequently implanted into the right hind flank of naïve mice. Seven days later, mice were challenged with viable CT26-EphA2 cells in the opposite flank, and the resultant tumors were evaluated for growth (Fig. 1E). As expected, mice primed with cisplatin-killed tumor cells exhibited tumor progression, consistent with the absence of ICD due to cisplatin, and therefore lack of vaccinal effects as previously demonstrated [45], whereas 7 of 10 animals receiving doxorubicin-treated cells rejected the tumor challenge. Importantly, 8 of 9 animals receiving photoimmunotherapy-treated cells rejected the new tumor challenge, further demonstrating that photoimmunotherapy is an ICD-inducing treatment that may augment vaccinal effects in the host.

### EphA2 photoimmunotherapy elicits innate and adaptive immune cell responses within treated tumors

**Fig. 2:**
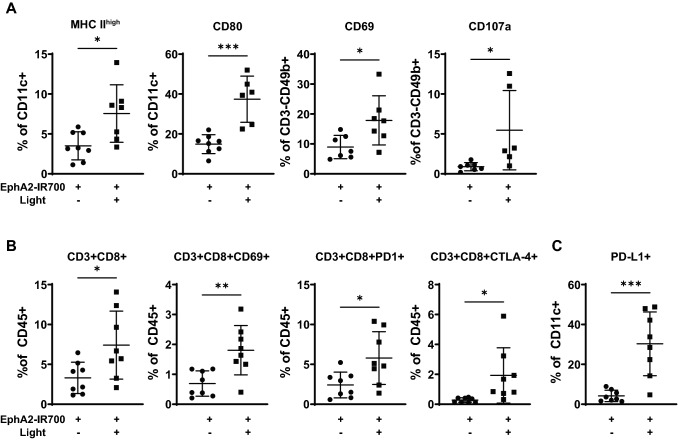
Photoimmunotherapy elicits intratumoral immune cell activation. Mice were implanted with CT26-EphA2 tumors, then treated with anti-EphA2-IR700 without illumination or EphA2 photoimmunotherapy. Activation markers for intratumoral innate and adaptive immune cells were measured by flow cytometry one day (**A**) or 7 to 8 days (**B & C**) after photoimmunotherapy treatment. *p < 0.05, **p < 0.01, ***p < 0.001 as measured by unpaired t-test

Photoimmunotherapy treatment in vitro induced cell surface expression and release of DAMPs, which led to DC activation and maturation (Supplementary Fig. S2 and S3). To characterize photoimmunotherapy-induced intratumoral immune responses in vivo*,* mice bearing CT26-EphA2 tumors received EphA2 photoimmunotherapy treatment, followed by tumor excision for flow cytometry analysis and characterization of intratumoral cells. Consistent with the in vitro observations, photoimmunotherapy treatment increased the frequency of DCs expressing the activation marker CD80, as well as those expressing high levels of MHC II, one day post treatment as compared to control mice that had received only anti-EphA2-IR700 in the absence of light (Fig. 2A). Moreover, photoimmunotherapy treatment increased the frequency of CD69, a marker of activation, and CD107, a marker of degranulation, expression on intratumoral natural killer cells. Seven to eight days after photoimmunotherapy treatment, the proportion of CD8 + T cells (of CD45 + intratumoral cells) was significantly increased, as well as the frequency of CD8 + T cells expressing markers of activation (CD69) or exhaustion (PD-1, CTLA-4) (Fig. 2B). Similarly, the frequencies of intratumoral CD11c + DCs displaying PD-L1, a cell surface marker expressed in response to IFN-γ exposure, were also significantly more abundant in photoimmunotherapy-treated mice (Fig. 2C). Together these data suggest that in vivo photoimmunotherapy treatment elicits profound and sustained activation of innate and adaptive immune cell responses within the TME.

### Photoimmunotherapy enhances anticancer activity of tumor-specific lymphocytes

**Fig. 3:**
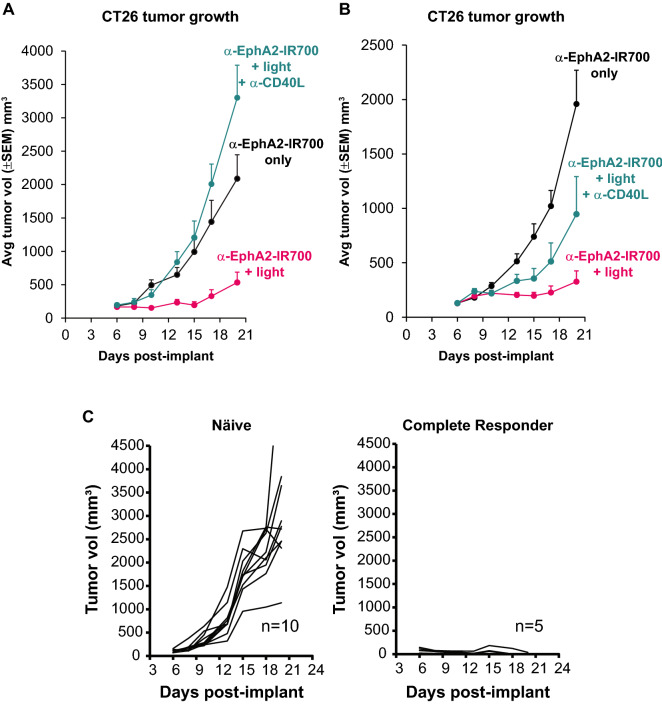
Photoimmunotherapy enhances anticancer activity of tumor-specific lymphocytes. Mice were implanted with CT26-EphA2 cells, then CD40L blocking antibody (or saline control) was administered at days 0, 1, 2 and 3 (**A**) or 6, 7, and 8. (**B**). Anti-EphA2-IR700 was delivered at day 6, followed by illumination 24 ± 2 h later for photoimmunotherapy groups, and tumor volume was measured to determine tumor growth inhibition (**A & B**). Final tumor measurements: EphA2 photoimmunotherapy vs EphA2 photoimmunotherapy + anti-CD40L, p < 0.01 (**A**) and n.s. (**B**) based on two-way ANOVA and Tukey test. To evaluate the memory potential against syngeneic tumors, mice identified as complete responders after photoimmunotherapy were rechallenged with syngeneic tumor cells in the opposite hind flank 49 days after the original tumor challenge, and 2 to 3 weeks after complete responses were achieved. Tumor growth was measured over 21 days as compared to growth in naïve mice (**C**). The tumor growth curves shown for both efficacy and tumor rechallenge are representative of at least two replicate experiments performed by different personnel

Pre-existing, tumor-specific CD8 + T cells significantly contribute to the antitumor activity of many immune modulating cancer therapies, and may also be significant for photoimmunotherapy [46]. To test this hypothesis, an anti-CD40L antibody that antagonizes the CD40:CD40L interaction to abrogate effector T cell priming was administered concurrently with tumor inoculation. Importantly, photoimmunotherapy treatment did not reduce tumor burden in mice that received anti-CD40L antibody administration, and thus lacked pre-existing immunity towards the tumor, when compared with mice that received photoimmunotherapy-treatment alone (Fig. 3A). As expected, mice that received anti-CD40L antibody administration without photoimmunotherapy treatment also exhibited significant tumor growth, similar to animals that received anti-CD40L administration with photoimmunotherapy (data not shown), due to the lack of adaptive immunity towards the tumor.

Because photoimmunotherapy increases DC activity in vitro and in vivo, photoimmunotherapy treatment may also augment the priming of new effector T cells for anticancer activity. To evaluate whether this phenomenon may occur, tumor-bearing animals were administered anti-CD40L at the same time as the photoimmunotherapy treatment, in order to suppress the possible priming of new effector T cells that could result from photoimmunotherapy treatment. Mice that received CD40L blockade at the time of treatment trended towards an impaired ability to inhibit tumor growth, despite photoimmunotherapy treatment (Fig. 3B). Mice that received anti-CD40L antibody administration without photoimmunotherapy treatment exhibited significant tumor growth similar to control animals (data not shown). Altogether these results indicate that both preexisting antitumor T cells and T cells primed upon photoimmunotherapy treatment play an important role in the antitumor efficacy of the photoimmunotherapy treatment.

Photoimmunotherapy treatment of CT26-EphA2 tumor-bearing mice resulted in 33% (5/15) of complete responses (CR), defined by the absence of detectable tumors at the end of the study (Fig. 3A). Complete responder mice were identified and rested for approximately 2 to 3 weeks before undergoing rechallenge with identical tumor cells injected into the opposite flank. Remarkably, all complete responder mice demonstrated complete tumor rejection at the distal site, suggesting that durable systemic adaptive immune memory responses were generated with initial photoimmunotherapy treatment (Fig. 3C). Taken together, these results indicate the ability of photoimmunotherapy treatment to augment the priming of tumor-specific T cells.

### Photoimmunotherapy combined with anti-PD-1 treatment leads to enhanced anticancer responses

**Fig. 4:**
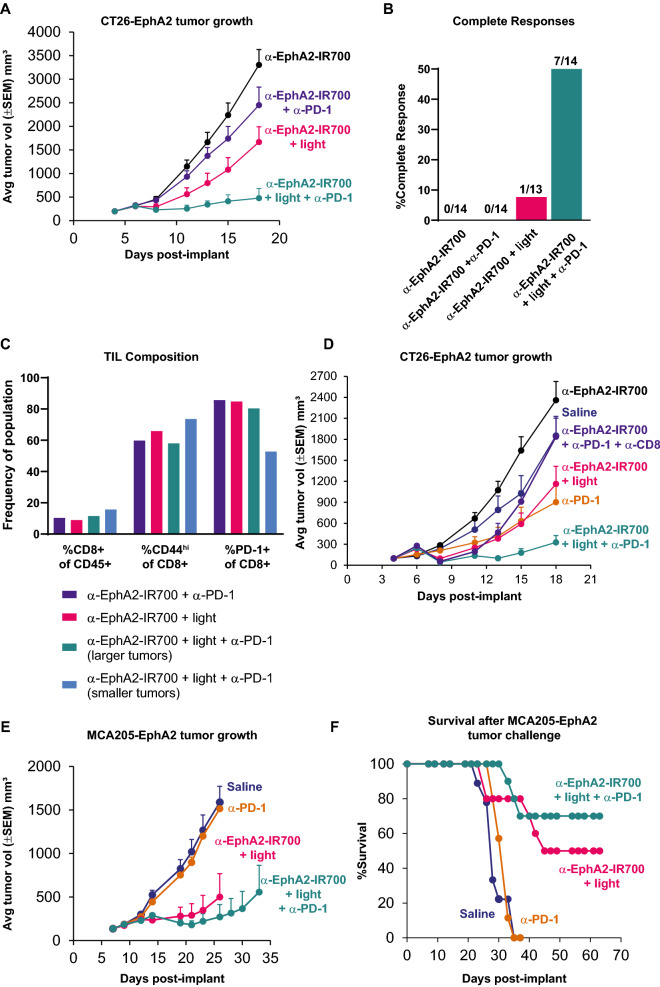
Combining photoimmunotherapy with checkpoint inhibition elicits enhanced anticancer responses. Mice were implanted with CT26-EphA2 tumors, then treated with anti-EphA2-IR700 alone, or EphA2 photoimmunotherapy. Anti-PD-1 was administered to indicated groups at days 4, 6, 8, and 11 post-inoculation, and tumor growth was measured from days 18 to 22, and graphed here through day 18 (**A**). Final tumor measurements: anti-PD-1 vs EphA2 photoimmunotherapy + anti-PD-1, p = 0.0001; EphA2 photoimmunotherapy vs EphA2 photoimmunotherapy + anti-PD-1, p < 0.05 based on two-way ANOVA and Tukey test. The percent of complete responses (calculated by number of CRs/total animals in treatment group) as defined as undetectable tumors at study end were determined (**B**). Activation patterns among intratumoral lymphocytes were determined by flow cytometry at day 21, among mice that had palpable tumors. Tumors with limiting volume were pooled to achieve sufficient cell yield for analysis (**C**). Mice were implanted with tumors (n = 15 per group) and received saline, anti-EphA2-IR700 without illumination, anti-PD-1 monotherapy (days 4, 6, 8, and 11 post implant), or anti-CD8 (days 4 and 7 for CD8 T cell depletion) and evaluated for tumor growth through day 18 post implant. Tumor-bearing mice also received photoimmunotherapy treatment (as described above, n = 12), photoimmunotherapy treatment with anti-PD-1 (n = 11), or photoimmunotherapy treatment with anti-PD-1 and anti-CD8 (n = 9), and evaluated for tumor growth through day 18. Final tumor measurements: EphA2 photoimmunotherapy + anti-PD-1 vs. EphA2 photoimmunotherapy + anti-PD-1 + anti-CD8, p < 0.01 based on two-way ANOVA and Tukey test (**D**). MCA205-EphA2 cells were inoculated into the hind flanks of mice. Mice were then treated with saline, anti-PD-1 alone, EphA2 photoimmunotherapy, or EphA2 photoimmunotherapy combined with anti-PD-1 antibodies, as indicated (n = 10 mice per group). Anti-PD-1 was administered beginning on day 7 and continuing 3 times per weeks until study end. Tumor growth (**E**) and survival (**F**) were measured through days 33 and 63, respectively. Final tumor measurements: anti-PD-1 vs EphA2 photoimmunotherapy + anti-PD-1, p < 0.0001; EphA2 photoimmunotherapy vs EphA2 photoimmunotherapy + anti-PD-1, n.s. based on two-way ANOVA and Tukey test (**E**). The tumor growth curves shown are representative of at least two replicate experiments performed by different personnel

Immune checkpoint inhibitor therapy, including anti-PD-1 agents that antagonize the immunosuppressive PD-1:PD-L1 interaction, has significantly improved clinical outcomes amongst patients suffering from many cancer types [1, 2]. Photoimmunotherapy treatment increased the frequency of CD3 + CD8 + PD-1 + T cells in the tumor and the expression of the ligand PD-L1 on dendritic cells (Fig. 2B & C). Therefore, CT26-EphA2 tumor-bearing mice were treated with photoimmunotherapy with or without anti-PD-1 therapy and were evaluated for tumor growth. As compared to previous experiments, here mice were implanted with an increased density of CT26-EphA2 tumor cells to model more aggressive tumors and better discern the combinatorial anticancer effects of anti-PD-1 with photoimmunotherapy. The combination treatment (EphA2 photoimmunotherapy plus anti-PD-1) elicited a notable reduction of tumor growth in comparison to each monotherapy treatment (Fig. 4A). Furthermore, 50% of mice that received combination treatment achieved CR, compared to photoimmunotherapy alone (7.7% CR) or anti-PD-1 monotherapy (0% CR) (Fig. 4B). The intratumoral CD8 + T cell responses from these mice were evaluated. In order to recover sufficient cell yield for analysis, tumors from mice that exhibited better responses (i.e., smaller tumors) to the combination of photoimmunotherapy and anti-PD-1 were pooled and compared to pooled tumors from mice with limited responsiveness (i.e., larger tumors) to that combination. The remaining groups of mice (anti-PD-1 monotherapy and photoimmunotherapy alone) were each pooled regardless of responsiveness. Dually-treated mice eliciting the most efficient antitumor responses exhibited an increased frequency of intratumoral CD8 + T cells, a marked upregulation of memory/activated CD44-expressing CD8 + T cells, and a reduced frequency of exhausted PD-1 + CD8 + T cells (Fig. 4C). To directly demonstrate the requirement of CD8 + T cells for anticancer responses resulting from the combination treatment, CT26-EphA2 tumor-bearing mice were dually treated with anti-PD-1 and photoimmunotherapy with or without CD8 + T cell depletion. CD8 + T cell-depleted mice that received dual therapies exhibited no tumor growth inhibition whereas immunocompetent mice receiving dual therapies exhibited robust anticancer responses (Fig. 4D). Because CD8 + T cell depletion completely abrogated anticancer activity, rather than only the anticancer responses provided by anti-PD-1, these results further indicate that photoimmunotherapy treatment augments the adaptive immune response and enhances the activity of anti-PD-1 agents.

To determine whether photoimmunotherapy can sensitize checkpoint inhibitor-resistant tumors to anti-PD-1 agents, mice bearing MCA205-EphA2 tumors (MCA205 tumors recombinantly expressing EphA2) received photoimmunotherapy alone, anti-PD-1 alone, or the combination treatment. As expected, anti-PD-1 monotherapy did not inhibit MCA205-EphA2 tumor growth, demonstrating its resistance to anti-PD-1 checkpoint therapy, whereas photoimmunotherapy treatment alone reduced tumor growth and extended survival. However, the combination treatment significantly reduced tumor growth and extended survival in comparison to anti-PD-1 monotherapy (Fig. 4E & F). Although not statistically significant, the combination treatment also trended towards enhanced complete responses and extended survival in comparison to photoimmunotherapy treatment alone. These results suggest that photoimmunotherapy treatment may sensitize anti-PD-1 resistant tumors to become anti-PD-1 responsive.

### Anti-PD-1 plus photoimmunotherapy treatment enhances systemic tumor-specific memory immune responses

**Fig. 5:**
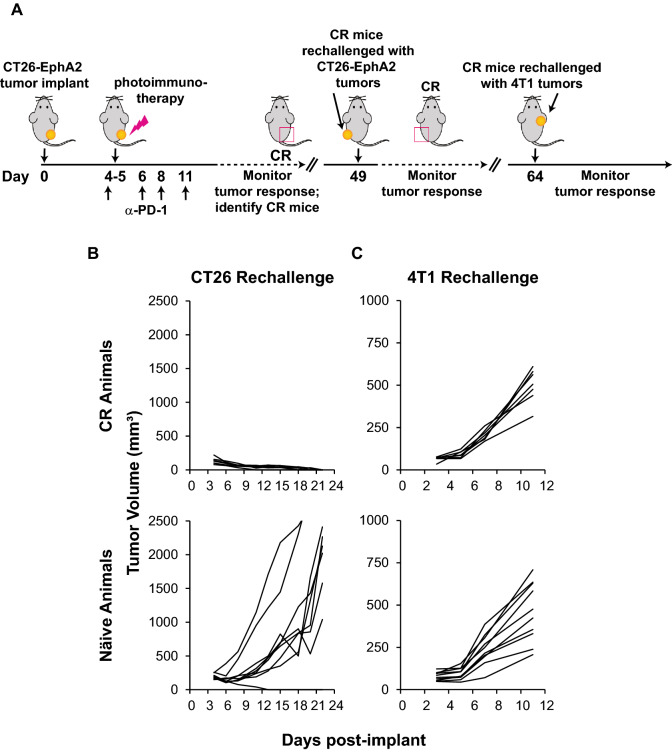
Complete responder mice, previously treated with anti-PD-1 and photoimmunotherapy, mount tumor-specific memory immune responses. Mice that had achieved a complete response (n = 7) after dual treatment with anti-PD-1 and photoimmunotherapy were re-challenged with syngeneic CT26-EphA2 cells in the contralateral hind flank, followed by 4T1 cells in the axilla (**A**). Naïve mice (n = 10) were inoculated as control. Tumor growth was measured to determine rejection (**B & C**). The resulting tumor growth curves after rechallenge are representative of at least two replicate experiments performed by different personnel

Mice that achieved complete responses after anti-PD-1 combined with photoimmunotherapy treatment were evaluated for their ability to combat new tumor challenges. At day 49 after the original CT26-EphA2 tumor implant, CR mice were re-challenged with syngeneic CT26-EphA2 cells in the contralateral hind flank, and tumor growth was monitored (Fig. 5A). All CR animals demonstrated a complete rejection of the secondary tumors whereas the naïve control animals experienced profound tumor proliferation (Fig. 5B). The same CR mice were then challenged with antigenically distinct 4T1 mammary tumor cells delivered to the right axilla at day 64 after the original CT26-EphA2 colon cancer implantation (Fig. 5A). CR mice did not exhibit heterologous protection against disparate 4T1 tumors since 4T1 tumor growth was similar in kinetics and volume to that of naïve mice (Fig. 5C).

### Photoimmunotherapy and anti-PD-1 treatment expands peripheral tumor-specific CD8 + T cells

**Fig. 6:**
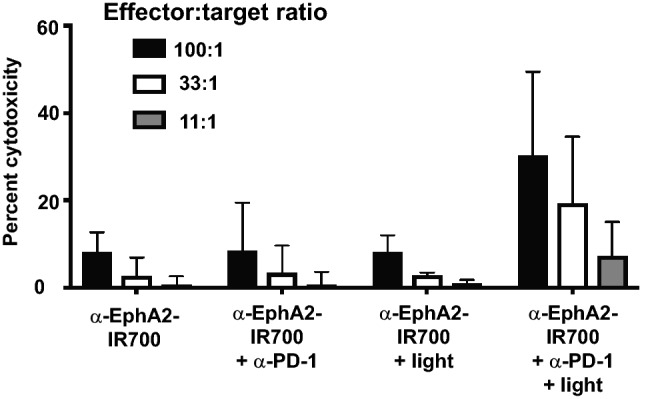
Photoimmunotherapy plus anti-PD-1 treatment expands peripheral tumor-specific CD8 + T cells. At day 14 after photoimmunotherapy, splenocytes from mice bearing CT26-EphA2 tumors that had been treated with either anti-EphA2-IR700, anti-EphA2-IR700 plus anti-PD-1, EphA2 photoimmunotherapy, or EphA2 photoimmunotherapy plus anti-PD-1, were harvested and expanded in the presence of the antigen AH1, which is expressed on the surface of CT26 cells, and co-cultured with CT26 cell monolayers in vitro at the indicated effector:target ratios. The AH1 antigen-specific cytotoxic potential of primed splenocytes was measured by Lactic Acid Dehydrogenase (LDH) release assay

To further support the antigen-specific cytotoxic potential of peripheral CD8 + T cells elicited by photoimmunotherapy plus anti-PD-1 treatment, splenocytes from CT26-EphA2 tumor bearing mice were harvested from the various treatment groups, expanded in the presence of the antigen AH1, which is expressed by CT26 cells, and then co-cultured with CT26 cells at effector:target ratios ranging from 100:1 to 11:1. Although the results were not statistically significant, there was a trend towards improved cytotoxic T lymphocyte (CTL) killing from mice that received dual photoimmunotherapy and anti-PD-1 treatment (Fig. 6). Taken together, these results suggest that dual treatment with photoimmunotherapy and anti-PD-1 generates durable antigen-specific immune memory responses (Fig. 5 & 6).

### Photoimmunotherapy plus anti-PD-1 combination enhances abscopal anticancer activity

**Fig. 7:**
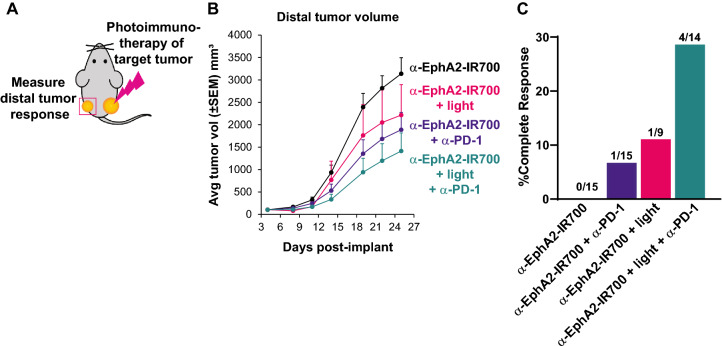
Photoimmunotherapy plus anti-PD-1 enhances abscopal anticancer activity. CT26-EphA2 cells were implanted into both hind flanks of mice. 3 × 10^6^ cells were implanted to establish primary tumors, whereas 3 × 10^5^ cells were delivered to establish distal tumors (**A**). Mice were treated with either anti-EphA2-IR700, anti-EphA2-IR700 plus anti-PD-1, EphA2 photoimmunotherapy, or EphA2 photoimmunotherapy plus anti-PD-1. Anti-PD-1 was delivered on days 4, 6, 8, and 11. Only target tumors, but not distal tumors were illuminated in groups receiving applied light. Tumor growth was measured through day 25 (**B**) and complete responses were determined at study end (**C**). The tumor growth curves shown are representative of at least two replicate experiments performed by different personnel

The induction of systemic, tumor-specific immune responses may elicit abscopal anticancer activity in distal, non-illuminated tumor lesions. To test this hypothesis, CT26-EphA2 cells were implanted into both hind flanks of mice with the distal, non-illuminated tumor receiving a comparatively reduced tumor cell burden to model metastatic disease (Fig. 7A). Mice were treated with photoimmunotherapy with or without anti-PD-1 administration, and only the target tumor, but not the distal tumor, was exposed to 690 nm light in groups receiving photoimmunotherapy (Fig. 7B). Consistent with a mechanism of enhanced systemic immunity, mice that received combinatorial photoimmunotherapy and anti-PD-1 demonstrated the highest rate of complete responses (defined in this case as absence of both target and distal tumors) (28.6%), and the most substantial distal tumor growth inhibition (TGI) (55.0%) as compared to photoimmunotherapy alone (CR 11.1%; TGI 29.4%) and EphA2-IR700 plus anti-PD-1 (CR 6.7%; TGI 39.8%) (Fig. 7C). Photoimmunotherapy treatment alone also appeared to mildly reduce distal tumor growth as compared to the control group. Taken together, these data suggest that photoimmunotherapy alone elicits mild abscopal effects which can be enhanced by the addition of anti-PD-1 treatment to provide a reduction in distal tumor volume.

## Discussion

Recently, broad enthusiasm has been directed towards harnessing the immune system to produce durable, self-perpetuating anticancer responses. The Cancer-Immunity Cycle, a concept whereby normal immune function becomes dysregulated in favor of promoting cancer, has informed key strategies for clinical intervention [47, 48]. Immune checkpoint inhibitors (ICI) antagonize the suppressive interactions between immune cells or tumor cells with effector T cells to reinvigorate T cell effector functions. Other strategies include vaccine approaches and targeting various T cell priming and trafficking molecules [47]. However due to intrinsic challenges for each treatment modality, the overall responses remain limited. For example, only approximately 20% of patients with solid tumors respond to ICI therapy, with high variability depending on tumor type, and accompanied with moderate to severe immunological toxicities that often arise from these ICI [3, 4, 49].

Photoimmunotherapy poses a promising treatment platform to combat tumors. Importantly, the unique mechanism of action whereby cell killing is not reliant upon biological signaling circumvents many hurdles and resistance mechanisms encountered by other approaches [8–16]. Tumors refractory to ICI therapy, such as anti-PD-1, may therefore retain sensitivity to photoimmunotherapy since the mechanism of action does not depend on the intratumoral immune contexture. Also, unlike radiotherapy or chemotherapy, tumor cell-specific destruction by photoimmunotherapy spares antitumor immune cells within the TME, and therefore does not risk compromising the hosts’ immune response against the tumor. Moreover, the enhanced release of cancer neoantigen elicits vaccinal effects [8, 16, 23, 39, 50, 51], harnessing the Cancer-Immunity Cycle and promoting a self-sustaining positive feedback loop (**Supplementary Fig S4**) [47, 48].

Along with studies performed by others [8, 24, 29, 30, 51], the results described in this publication demonstrate that cancer killing by photoimmunotherapy also enhances antitumor immune responses. Photoimmunotherapy treatment resulted in the immediate disruption of the tumor cell membrane for cell lysis and induction of immunogenic cell death, characterized by the release of DAMPs. Dendritic cells proximal to the targeted cancer cells upregulated co-stimulatory receptors and secreted pro-inflammatory cytokines after 690 nm illumination, supporting a direct innate immune response to photoimmunotherapy treatment. Furthermore, effector CD8 + T cells and NK cells expressing CD69, which is associated with positive clinical outcomes, were enriched in treated tumors [3, 6, 52–56]. T cell responses induced by photoimmunotherapy were not restricted to the TME however— peripheral CD8 + T cells were also generated and exhibited potent anticancer activity to inhibit the growth of newly inoculated tumor cells at a location disparate to the original tumor site.

Anticancer responses by pre-existing tumor-specific immune cells also significantly contributed to efficacy in mouse models. Although tumor neoantigen exposure was not specifically studied here, tumor rejections elicited by complete responder mice, as well as reduced efficacy of photoimmunotherapy among mice that received CD40/CD40L blockade, suggest that photoimmunotherapy enhances the expansion of tumor-specific immune responses. Moreover, although this study did not formally demonstrate the T cell receptor (TCR) repertoire generated by photoimmunotherapy, we surmise that this approach may generate a diverse polyclonal pool of tumor-specific T cells, which would provide further opportunity for polyfunctional responses and also contribute to driving the Cancer-Immunity Cycle.

Photoimmunotherapy may also be combined with immunomodulatory strategies to enhance the anticancer response. The combination of anti-PD-1 with photoimmunotherapy treatment resulted in striking suppression of tumor growth both in primary tumor challenge and syngeneic re-challenge experiments. Anticancer responses included activated CD44 + CD8 + T cells which accumulated in the TME. Moreover, using an anti-PD-1 ICI resistant tumor model of mice, this study further demonstrated that combinatorial anti-PD-1/photoimmunotherapy treatment can overcome anti-PD-1 resistance mechanisms to inhibit tumor growth and improve survival outcomes.

Finally, the potential for anti-PD-1 combined with photoimmunotherapy treatment to elicit abscopal effects was determined. In that context, the combination treatment enhanced the growth inhibition of the distal tumor when compared to photoimmunotherapy alone. This included complete responses at the distal site generated in a higher percentage of mice receiving combination treatment. These results further support that photoimmunotherapy combined with immunomodulators enhances systemic anticancer immunity and may be utilized to improve clinical responses.

Indeed, cancer targeted photoimmunotherapy treatment has been evaluated in a Phase 1/2a study conducted in the United States (Study of RM-1929 and Photoimmunotherapy in Patients With Recurrent Head and Neck Cancer, clinicaltrials.gov NCT02422979) [57], and a Phase 1 study in Japan (A Phase I single Center, Open-Label, Combination study of RM-1929 and Photoimmunotherapy in Patients with Recurrent Squamous Cell Head and Neck Cancer) [58]. Both studies demonstrated a manageable safety profile, with clinically meaningful responses and favorable survival rates in patients with recurrent head and neck squamous cell carcinoma (rHNSCC) who had previously failed several lines of treatment, including anti-PD-1 therapy in some cases [57, 58]. Two additional trials are currently enrolling to: i) evaluate the use of cetuximab-IR700 photoimmunotherapy in heavily pretreated rHNSCC patients (global Phase 3 trial: ASP-1929 Photoimmunotherapy (PIT) Study in Recurrent Head/Neck Cancer for Patients Who Have Failed at Least Two Lines of Therapy; clinicaltrials.gov NCT03769506), and ii) determine the utility of combinatorial anti-PD-1 with cetuximab-IR700 photoimmunotherapy for treatment of recurrent and/or metastatic HNSCC, and locally advanced or metastatic cutaneous SCC (Phase 1b/2 trial in the United States: An Open-label Study Using ASP-1929 Photoimmunotherapy in Combination With Anti-PD-1 Therapy in EGFR Expressing Advanced Solid Tumors; clinicaltrials.gov NCT04305795). Moreover, these results fueled Federal Drug Administration Fast Track Designation for the cetuximab-IR700 product and laser device, ASP-1929 photoimmunotherapy, in the United States in 2018, and marketing approval by the Pharmaceuticals and Medical Devices Agency (PMDA) in Japan in late 2020.

Thus, photoimmunotherapy poses a unique opportunity for the treatment of solid tumors by causing tumor cell-specific membrane disruption without collateral tissue damage, and eliciting durable and tumor-specific immune responses. Moreover, systemic toxicity is minimal in comparison to other tumor cell targeting modalities, the platform is adaptable to any target receptor of interest [32], and other immunotherapy approaches may be used in combination for enhanced efficacy, even in the instance of ICI-resistant tumors. These results demonstrate that cancer-targeted photoimmunotherapy elicits tumor-specific immune responses that may be augmented when combined with immunomodulators.

### Supplementary Information

Below is the link to the electronic supplementary material.

Supplementary file1 (PDF 1609 KB)

## Data Availability

Data sharing is not applicable to this article as no datasets were generated or analyzed during the study.
