# Supplementary material for: Cancer-targeted photoimmunotherapy induces antitumor immunity and can be augmented by anti-PD-1 therapy for durable anticancer responses in an immunologically active murine tumor model
Source: Cancer Immunol Immunother. 2022 Jul 1;72(1):151–68. doi: 10.1007/s00262-022-03239-9 (PMC9813181; doi:10.1007/s00262-022-03239-9)
Supplement: Supplementary file 1 — Supplementary file1 (PDF 1609 KB) [file 262_2022_3239_MOESM1_ESM.pdf]

Hsu *et al* Supplement

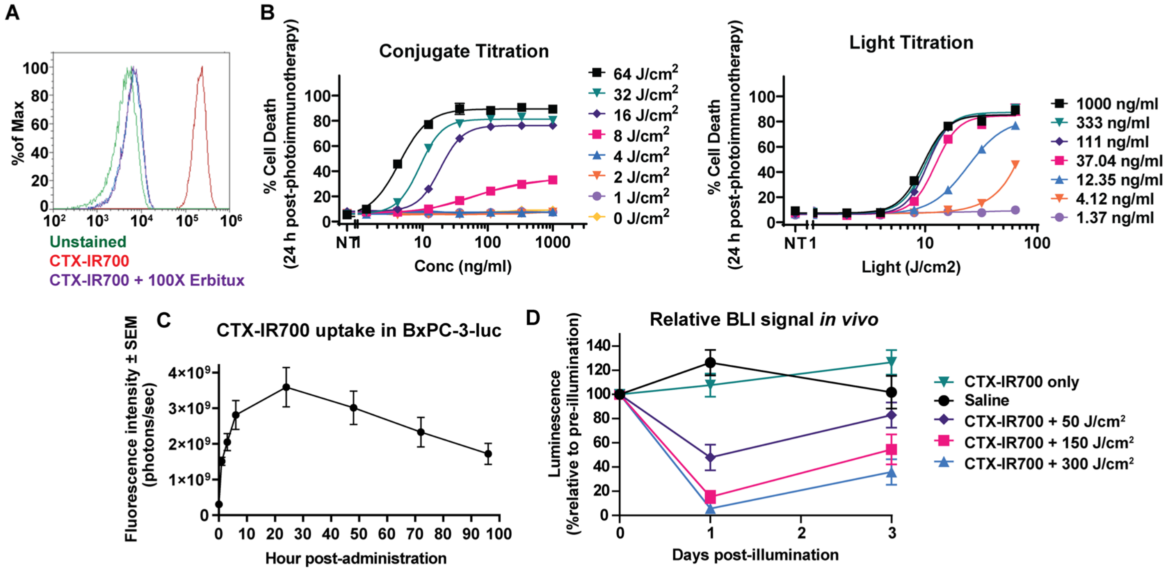

Supplementary Figure 1. Cetuximab-IR700 binds EGFR on tumor cells and induces rapid cell death after light exposure *in vitro*. CTX-IR700 or CTX-IR700 plus 100-fold excess anti-EGFR antibody (CTX) was added to BxPC-3 cells *in vitro*. Binding was evaluated by flow cytometry (**A**). CTX-IR700 was added to BxPC-3 cells in increasing concentrations. Next, light was applied at indicated doses to determine dose dependency of both antibody-conjugates and applied light (**B**). CTX-IR700 conjugate uptake into the tumor was evaluated by measuring IR700 fluorescence within the tumor over time (**C**). Nude mice were challenged with BxPC-3-luciferase-expressing tumor cells, then saline or a single dose of CTX-IR700 with or without applied light was delivered. Light was applied one day later. Bioluminescence, as a measure of viable tumor mass, was measured at days 1 and 3 after light application (**D**).

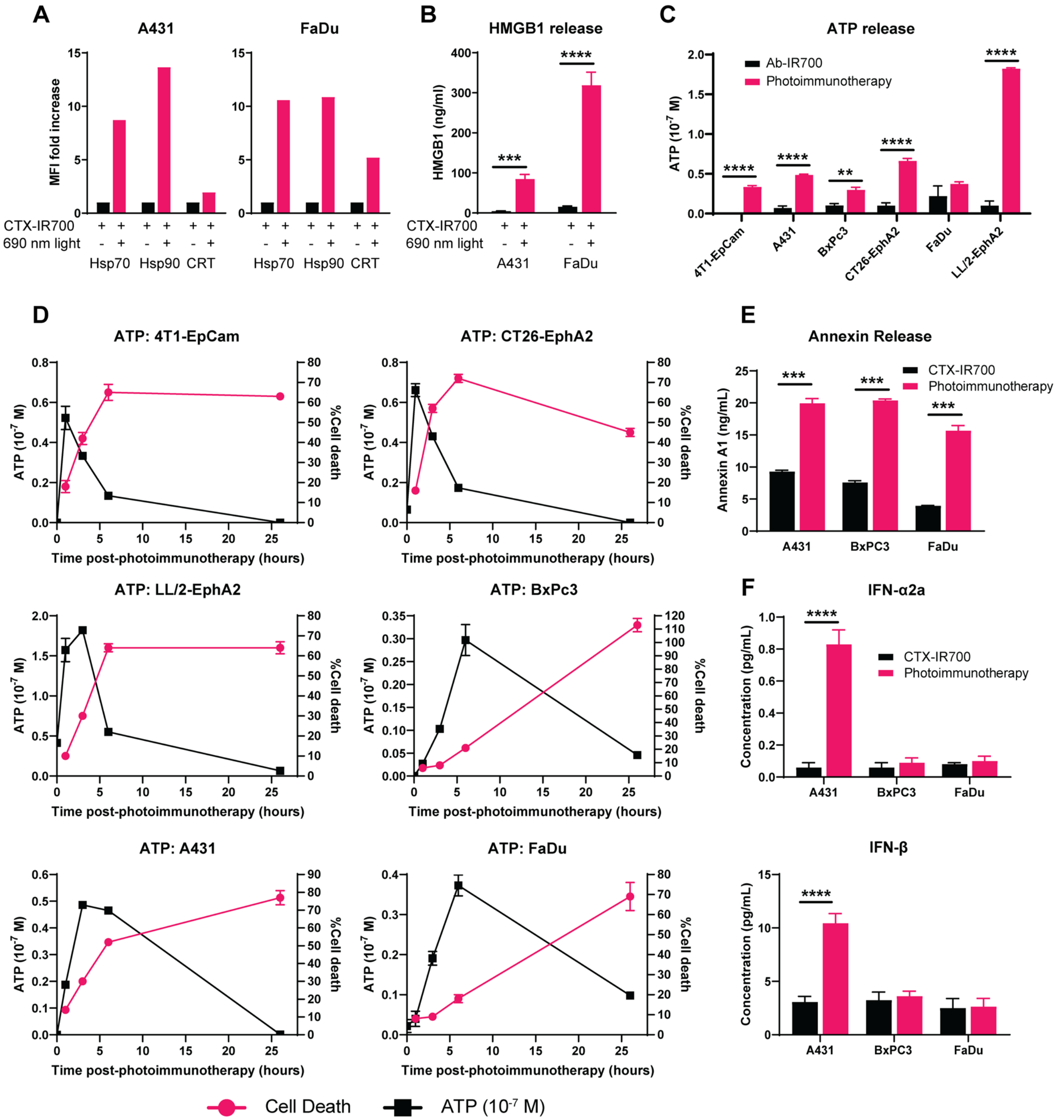

Supplementary Figure 2. *In vitro* photoimmunotherapy induces the release of ICD molecules. A431 or FaDu cells were treated with CTX-IR700 only, or CTX-IR700 photoimmunotherapy. CTX-IR700 photoimmunotherapy-treated carcinoma cells demonstrated a trend toward an increase in surface expression of Hsp70, Hsp90 and CRT as observed by flow cytometry (**A**), and a significant increase in soluble HMGB1 in culture supernatants (**B**). ATP release following photoimmunotherapy across multiple carcinoma cell lines and target antigens was measured using an ATP Bioluminescent Assay Kit (Sigma-Aldrich) (**C**). In all tumor cell lines tested, the peak of ATP release occurred within 2 to 6 hours following photoimmunotherapy and

preceding maximal cell death, as measured by lactate dehydrogenase (**D**). Cultured A431, BxPC-3, or FaDu cells were treated with CTX-IR700 photoimmunotherapy or antibody-conjugate alone. Culture supernatants were assayed for secreted Annexin A1, IFN-α2a, and IFN-β using the MSD platform (**E & F**). **p<0.01, ***p<.001, ****p<0.0001 as measured by unpaired t-test.

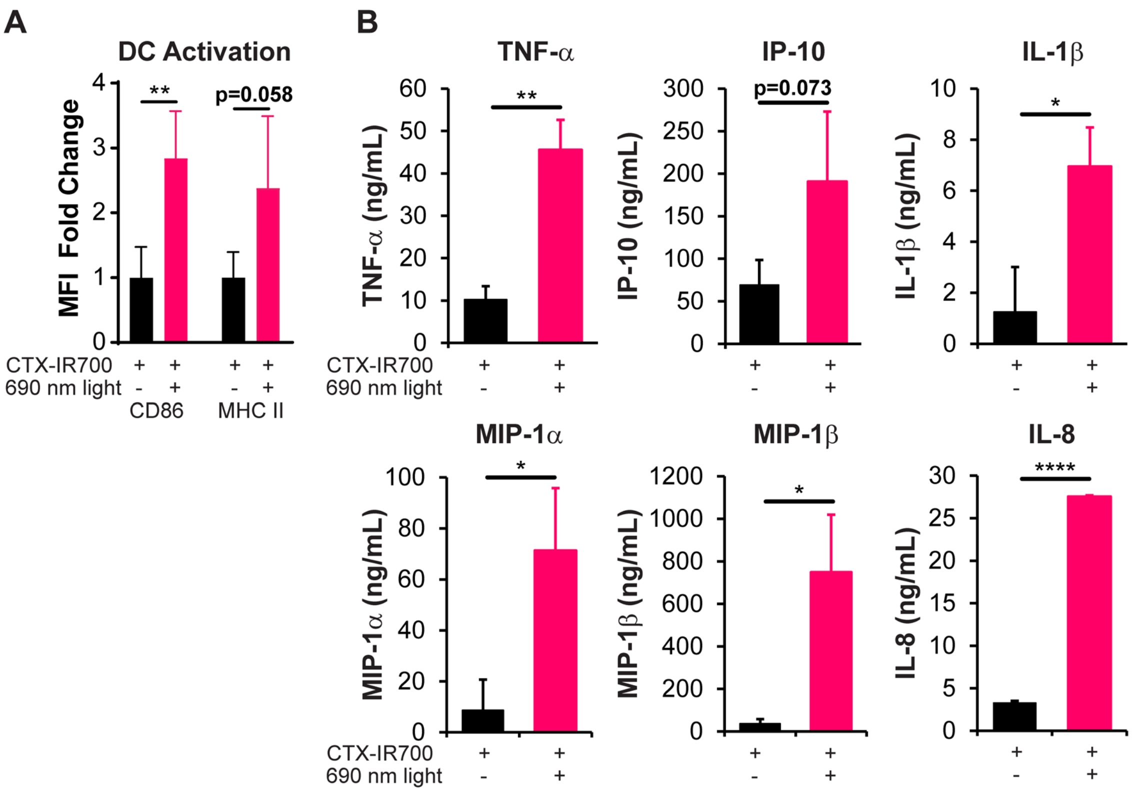

Supplementary Figure 3. Human DCs exposed to photoimmunotherapy-killed cancer cells become activated. Photoimmunotherapy-killed or untreated control FaDu cells were co-cultured with primary iDCs derived from 4 individual healthy human donors for 2 days for flow cytometry analysis (**A**) or cytokine analysis (**B**). After indicated times, cells were gently detached and stained for DC activation markers (**A**), and supernatants were collected for cytokine analysis by Luminex. Results from n=3 technical replicates are shown (**B**). Statistics were calculated with an unpaired t-test per cytokine. *p<0.05; **p<0.01; ****p<0.0001.

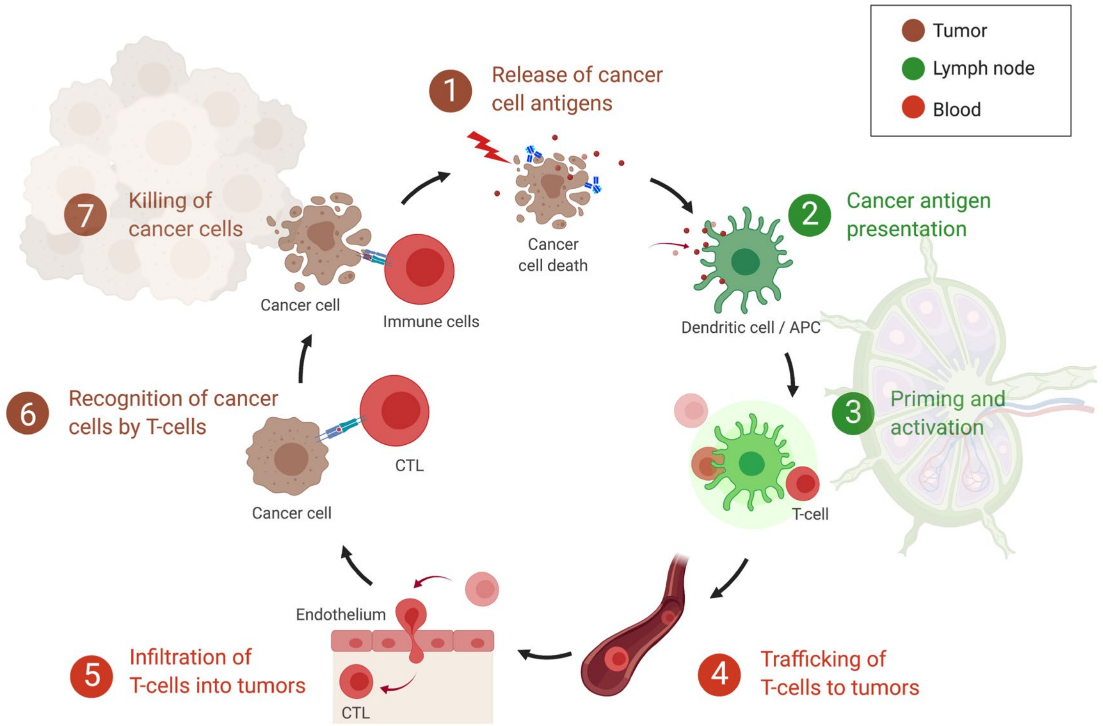

Supplementary Figure 4. Proposed immune mechanisms initiated by photoimmunotherapy. Photoimmunotherapy induces rapid tumor cell death, releasing cancer cell antigens consistent with immunogenic cell death. Local antigen presenting cells respond by antigen uptake and upregulation of co-stimulatory receptors for T cell activation. Cancer antigen-specific effector T cells further target cancer cells, allowing continued immune stimulation and fulfilling the Cancer-Immunity Cycle. Created with BioRender.com.

Supplementary Table 1. Flow cytometry reagents.

Reagent	Company	Cat. No.	Clone
Zombie NIR Viability Dye	Biolegend	431111	
**Human**			
CD11c PeCy7	BD Biosciences	561356	B-ly6
HLA-DR APC/Fire750	Biolegend	307658	L243
CD86 PerCP Cy5.5	Biolegend	305420	IT2.2
Mouse IgG1 PeCy7 (isotype)	BD Biosciences	557872	MOPC-21
Mouse IgG2a APC/Fire750 (isotype)	Biolegend	400284	MOPC-173
Mouse IgG2b PerCP Cy5.5 (isotype)	Biolegend	400338	MPC-11
**Mouse**			
CD45 PeCy7	Biolegend	103114	30-F11
CD3 FITC	Miltenyi	130-109-836	REA641
CD69 PerCP-Vio700	Miltenyi	130-103-945	H1.2F3
CD8 APC	Miltenyi	130-109-248	REA601
CD3ε VioBright FITC	Miltenyi	130-109-246	REA606
CD44 PE	Biolegend	103008	IM7
CD107a PerCP-Cy5.5	Biolegend	121626	1D4B
CD49b PE	Miltenyi	130-120-885	REA541
MHC II PerCP-Cy5.5	Biolegend	107626	M5/114.15.2
CD11c AlexaFluor 488	Biolegend	117311	N418
CD11c PerCP-Cy5.5	Biolegend	117328	N418
CD80 PE	Biolegend	104722	16-10A1
CTLA-4 PE	Biolegend	106306	UC10-4B9
PD-1 PerCP-Vio700	Biolegend	135208	29F.1A12
PD-L1 PE	eBioscience	12-5982-82	MIH5
Rat IgG2a, κ-PerCP/Vio700 (isotype)	Biolegend	400530	RTK2758
Rat IgG2b, κ-PE (isotype)	Biolegend	400607	RTK4530
